# Inlay Cavity Preparation

**Published:** 1904-05-15

**Authors:** F. E. Roach


THE
DENTAL REGISTER.

Vol. LVIII. MAY 15, 1904. No. 5.

### INLAY CAVITY PREPARATION.

BY F. E. ROACH, D.D.S.

Read before the Southwestern and Central Michigan Dental Societies, April 13, 1904.

Having taken a radical stand in regard to cavity preparation for porcelain inlays and in view of the very prevalent idea that cement adhesion affords sufficient retention, I thought when asked to give you a paper on porcelain inlays, that this phase of the subject would probably be of some interest to you, and while I do not know that I can offer anything especially new, it is my hope that what I shall say may elicit a discussion that will prove beneficial to us all.

The repid development and high state of perfection of the porcelain inlay of to-day would seem to warrant the ever-increasing confidence in its permanence, and its near approach to the ideal places it in the front rank as a filling material for a certain class of cavities.

While possessing, at present, an inherent friability which precludes it from first place in cavities exposed to masticatory forces, it will, however, prove eminently satisfactory, when properly inserted in judiciously-chosen cavities. The practitioner who fails to give himself the use of this new filling material is doing himself and his patients an injustice. He not only deprives his patients of the benefits of one of our most valuable aids in tooth restoration and preservation, but he likewise deprives himself of a really pleasant and profitable part of dentistry.

To attain any degree of success in this line of work, it is imperative that each step be taken with precision and a careful observation of minor and seemingly-insignificant details.

It is my belief that the same basic principles involved in the preparation of cavities for the metallic fillings should apply as well to cavities for inlays. The cavity preparation for the various filling materials demands a slight modification to suit certain characteristic features peculiar to each. While the preparation for the porcelain inlay is somewhat different from that of gold and amalgam, the principles of "convenience, form, seating, anchorage, extension for prevention, definite margins," etc., should apply with equal force.

It is claimed by some of the recognized experts in this work that close adaptation and crystallization of the cement under pressure are the only requirements for retention. Having satisfied myself by experimentation and clinical observation of the fallacy of such practice, I am endeavoring to work out a system of cavity preparation founded on mechanical principles.

It is not my intention, nor is it possible in this short paper, to present more than a superficial view of the subject.

Without entering into a discussion of the various methods that are in use at present, I will pass at once to a consideration of my system, which, though incomplete, is nevertheless a system and will apply in a general way to most all cavities.

In leading up to the subject proper and that you may better understand my classifications, I will indicate briefly and in a general way when and where porcelain is indicated.

In a broad sense porcelain may be indicated in any conspicuously located cavity, where sufficient bulk of material and secure anchorage is obtainable. More specifically porcelain is indicated, in conspicuously located cavities for æsthetic reasons; in teeth lacking in structural strength porcelain will render the most permanent service; when the nervous strain incident to a proper condensation of gold is obviously to be avoided; and in teeth loosened by disease or otherwise incapacitated to endure gold filling.

*Contra-indications.*—Porcelain is contra-indicated in small and inconspicuous cavities where gold or amalgam may be used to advantage, and in large restorations in molars and bicuspids where the gold inlay meets every requirement except color, and will better withstand the strain of mastication.

*Advantages.*—Among the advantages of porcelain over gold or amalgam may be mentioned harmony of color, compatibility, permanence of form and volume, greater immunity to recurrent decay and less exhaustion to both patient and operator during insertion. Its principal disd vantage is its inherent friability.

First, we may have a classification of cavities in the order of their favor for porcelain. 1st, Labial; 2nd, Gingival; 3rd, Proximal cavities in incisors and cuspids; 4th, Buccal; 5th, Incisal; 6th, Proximo-incisal; 7th, Occlusal; 8th, Mesio-proximo-occlusal; 9th, Disto-proximo-occlusal.

Second, we may have a classification of cavities with reference to their requirements for retention as follows:

1st. Cavities in which frictional resistance affords sufficient retention. Namely, labial, buccal, simple occlusal and proximal caviiies.

2nd. Cavities in which direct plans of resistance are required. Namely, proximal cavities that include incisal and occlusal angles, the walls of which are sufficiently strong to afford adequate means of retention.

3rd. Cavities in which some form of pin anchorage is necessary. In this class we may include all large restorations. Labial inlays are first in favor because of their conspicuous location, simple construction and freedom from masticatory forces.

Labio-gingival inlays occupy the same position regarding stress, but being less conspicuous and more difficult of construction are given second place.

Proximal inlays in incisors and cuspids are among the most conspicuous, but being more difficult of construction are given third place.

Buccal inlays for cavities, except in second and third molars, are not difficult to make and are free from stress, but owing to their inconspicuous location are fourth in favor.

Incisal restorations, while hazardous risks in some cases, are usually justifiable for æsthetic reasons and are placed fifth in favor.

Proximo-incisal restorations are quite as conspicuous and hazardous and being more difficult are given sixth place.

Occlusal inlays being inconspicuous and hazardous are given seventh place.

Proximo-occlusal restorations are in many cases very conspicuous and justifiable but are given eighth place, being extra hazardous and difficult to make, are seldom permissible and occupy ninth and last place on the list.

*Cavity Preparation.*—The preparation of cavities for the reception of the various filling materials demands a slight modification to suit certain characteristic features peculiar to each material, and, while the preparation for the porcelain inlay is some what different from that of gold and amalgam, the fundamental principles apply to all alike, namely, free access, extension for prevention, secure seating and anchorage, definite margins and so forth.

After breaking down frail walls and removing all decay, use a cross-cut fissure bur and with a sweeping motion straightened the walls and when the general outline is satisfactory should there still remain undercuts, they should be filled with cement after which the entire surface should be gone over carefully and made smooth so that no obstruction will be left to interfere with drawing the matrix. This is best accomplished with the diamond fissure bur or a similarly shaped gem or Arkansas stone.

To obtain the best results in this work, the following rules should be closely observed:

1st. Obtain free access.

2nd. Cavity walls should slightly diverge from base to orifice in the direction the matrix is to be drawn.

3rd. The margins must be sharp and well defined and not beveled.

4th. Avoid all undercuts.

5th. Cavity must be so shaped that some form of anchorage will supplement cement adhesion.

Unless free access is obtained the matrix cannot be properly burnished, withdrawn, nor the inlay inserted.

Diverging walls facilitate removal of matrix and insure a better fitting inlay.

Margins must be definite in order to get good joints and continuity of inlay with tooth. A bevel will yield an inlay with a feather edge, which is always an element of weakness and must be avoided.

It is impossible to draw a matrix where undercuts exist. Cut them out or fill with cement.

While it is not impossible to produce sharp angles in porcelain, it is easier and more artistic, if the general outline of the cavity is straight lines merged with rounded corners.

All inlays must have one or more of the following means of anchorage supplementing that of cement adhesion; either positive or frictional planes of resistance or some form of pin anchorage. By positive planes of resistance, we mean self-retentive form of cavity, such as the dovetail or its equivalent. The straight mortice-joint or the builders' lap of the brick serve as an example of frictional resistance for anchorage. By pin anchorage, we mean baking a pin into the inlay to correspond with a hole drilled into which the pin is cemented for anchorage.

In conclusion, I would emphasize the importance of a careful study of this phase of the subject as it is the foundation of successful inlay work.

DISCUSSION.

Dr. W. T. Reeves: The essayist has no occasion for introducing his paper with an apology. He has given us a very satisfactory classification. I had intended taking exceptions to that portion of the paper which appeared to indicate that it was necessary in inlay work of porcelain to provide cavities of retentive form, but the closing paragraph clears the matter so that any remarks on that subject would seem unnecessary.

It is not necessary that cavities be prepared with undercuts and other retentive forms to secure frictional resistance. Close adaptation will provide frictional resistance whatever may be the cavity form. We should have parallel walls when they will permit of proper manipulation to secure a matrix and an inlay that fits perfectly. A perfect fit properly cemented gives perfection of resistance to disturbing stress. A seating form of wedge shape is desirable as it gives positive direction when setting the inlay and secures closer adaptation. A symmetrical form is easily misplaced and allows of inaccurate adaptation. All margins should be beveled inwardly, that is to say they should present a well-defined or knife edge. This kind of a margin will give an obtuse angle to the inlay margin and prevent its fracturing under percussion, or bleaching out the color in firing. Porcelain inlays are friable when the margins are then they are often made friable by faulty firing. If the porcelain is overbaked it becomes brittle. In molar and bicuspid inlays for proximo-occlusal cavities, get as much bulk as possible in the inlay and do not fire to a full glaze, just enough to prevent adhesion of food stuffs, etc. I do not believe in baking pins into inlays, as they weaken the porcelain more than they give retentive security. A thin lap-joint will be more likely to slip than a square butt-joint It may seem strange to hear it, but in my experience I find that inlays which are subject to stress stand better than labial, buccal, or occlusal inlays having surrounding tooth walls. I don't know why this is so, but I have more failures of labial and buccal inlays than all others. It may be that the cements do not get a chance to set so firmly as in other places.

DR. H. D. WATSON: In my experience I have found these buccal and lingual inlays more likely to fail than any others, as Dr. Reeves has said. I have attributed the cause to the fact that it is not possible to insert the inlay under pressure as we can do in many other cases.

DR. W. H. CUDWORTH: I am inclined to believe that success in this work depends very largely on the cavity preparation which takes into account retentive form of the cavity. We can not safely depend on the close adaptation and cement in cases where stress comes on the inlay. Much injury to the cause of porcelain inlay work comes from the statements that retentive form is unnecessary, and from the saucer-shaped cavities made in clinics and by commercial exhibitors. So many failures are certain to follow such ideas that many will be dissuaded from perfecting the technical details which are necessary to do this work acceptably. Pin retention is only advisable in restoration of corners or in making tips at exposed places. In proximo-incisal cavities of the front teeth, make a seat at the cervical wall with a suitable inverted cone bur, and and then with a special-pointed or cone-shaped bur groove the labial or lingual wall so that the matrix can be made and removed and the inlay will wedge to place. In these cavities no saucer preparation will sufficiently anchor an inlay, no matter how close the adaptation may be. Inlays made of porcelain, unless very thin, should not be friable; they are harder and more resistant than any metal fillings. I suspect that in making labial and buccal inlays we do not take the same care as in those cases which seem more liable to be disturbed, consequently we lose more of these inlays. We don't know as much about this subject as we shall. Our methods are as yet not settled to definite procedures. We expect to exercise as much latitude as we do in metal work. The personal equation also cuts as much of a figure here as in metal filling. In regard to bodies, I use the Jenkins body and believe it the only one fusing below pure gold that should be used at all. I use it with the platinum matrix and not with the gold and think I get better results. I use the platinum matrix because I can burnish it to the cavity as easily as gold, and it stays when worked to the shape of the cavity much better than gold. I can also fire it without investing if I choose, and take less risk of fusing it and spoiling my work. I think it also gives the closest adaptation when carefully burnished.

Dr. Reeves: As Dr. Cudworth has well said we are all learning, and I should be glad to answer any questions about this work that I can. We want to get the experiences of each other that we may compare notes, as by so doing we shall be stimulated to progress. Porcelain inlay workers are universally known as willing to give up their secrets, this is because of the high esteem in which we hold this work and the confident hope we entertain that it will in the near future take the place of much of the present metal filling.

Dr. Cudworth: One of the serious disadvantages of this work is in the furnace work; we have now good furnaces of all kinds, but the bright light of the furnace is very trying to the eyes, especially when continuously done. We should encourage manufacturers to produce for us inexpensive pyrometers that our eyes may be saved. There is one advantage of low-fusing bodies, and that is that the heat required is not so intense but one can look at it with little damage to the eyes. In mixing the porcelain powders I use preferably alcohol, as the excipient. The batter can be made quite thin and it will flow into every part of the matrix, the alcohol can then be evaporated and the porcelain powder will be solidly packed into the matrix and when fused it will be dense.

Dr. Reeves: I believe in mixing the porcelain powder with water and using it as dry as it is possible to pack it. By jarring the pliers which hold the matrix with a roughlyknurled steel instrument handle in a sawing-like motion, you will bring the water to the surface and at the same time settle the body into the matrix in a very solid form, in fact it will be more dense than if done under great pressure. The moisture which comes to the surface should be taken up by the addition of dry powder, and not with absorbent paper.

DR. ROACH: I would in closing emphasize the necessity of cavity preparation with all the retentive form practical. Labial and buccal inlays often fail because the side walls diverge excessively; they should approach the parallel as much as possible. In this way we get the most mechanical retention by frictional adhesion. I do not place much value on the dry mixing of the porcelain or on whether you jar the material into the matrix by tapping or by the sawing effect of a rough instrument handle. This is a technical detail of a personal character only.

